# The prevalence of xerostomia among e-cigarette or combustible tobacco users: A systematic review and meta-analysis

**DOI:** 10.18332/tid/156676

**Published:** 2023-02-09

**Authors:** Xingtong Guo, Lili Hou, Xuepei Peng, Fuyou Tang

**Affiliations:** 1 School of Nursing, Chengdu University of Traditional Chinese Medicine, Chengdu, China; 2 Nursing Department, Shanghai Ninth People’s Hospital, Shanghai Jiaotong University School of Medicine, Shanghai, China

**Keywords:** e-cigarettes, meta-analysis, tobacco, xerostomia

## Abstract

**INTRODUCTION:**

This study aims to evaluate the prevalence of xerostomia in a healthy population with e-cigarettes and/or combustible tobacco.

**METHODS:**

The following electronic databases were searched: Web of Science, Chinese Biomedical Literature Database (CBM), PubMed, Cochrane Library, Embase, Chinese National Knowledge Infrastructure (CNKI), China Science and Technology Journal Database (VIP), and Wan-Fang Database, from 1 January 2000 to 20 October 2022. The language was limited to Chinese and English. The data were analyzed using Stata 15.0, and the prevalence of xerostomia in different smokers is reported.

**RESULTS:**

A total of 14 studies were included, with a total sample size of 6827 cases. The overall prevalence of xerostomia was 26% (95% CI: 18–35). In the combustible tobacco population, the prevalence of xerostomia was 24% (95% CI: 21–27), while among e-cigarette users it was 33% (95% CI: 18–48).

**CONCLUSIONS:**

Current evidence suggests that the prevalence of xerostomia is high in healthy smoking populations. These findings are restricted by the number and quality of the included studies and need to be validated by additional high-quality studies.

## INTRODUCTION

Nearly 1 billion individuals worldwide suffer from the health risks associated with smoking tobacco^1^. The World Health Organization (WHO) states that in 2004, tobacco use caused the deaths of nearly 5 million people aged ≥30 years, worldwide^2^. CDC analyzed data from the 2019 National Health Interview Survey (NHIS) in 2019, and estimated that 50.6 million US adults (20.8%) reported currently using any tobacco product, including combustible tobacco (14.0%) and e-cigarettes (4.5%). E-cigarette use was highest among adults aged 18–24 years (9.3%)^3^. According to a study in the New England Journal of Medicine, the number of adolescents using e-cigarettes increased by 10% between 2017 and 2018, affecting 1.3 million young people, and continued to rise from 2018 to 2019^4,5^.

It is well known that smoking is a significant factor for oral health problems. Smokers are exposed to more than 7000 chemicals per puff, which alters the salivary component, resulting in impaired oral protection^6^. Seeme et al.^7^ showed that active smokers had higher clinical oral dryness (COD) scores and lower salivary flow rate (SFR). Xerostomia, a subjective sensation of dry mouth, is a side effect of e-cigarette use in adults and youth^8,9^. Lack of saliva secretion can lead to difficulties swallowing, chewing, and speaking, as well as mouth burning, poor breath, altered taste, oral mucosa dryness, tongue inflammation, cracked and flaking lips, oral candidiasis, and dental caries, resulting in poor quality of life for the people^10,11^. Xerostomia affects 5.5% to 46% of the population, most usually older, and is more common in women than men^12^. Additionally, 9% of Australians between the ages of 15 and 34 years, have reported experiencing dry mouth which may harm young people^13^. The prevalence of xerostomia has been related to a worse level of oral health-related quality of life in a study of people in their early 30s^14^. People who lack saliva are at an increased risk of oral infection and dental caries^11^.

Combustible tobacco and e-cigarette use may be more prevalent among the risk factors of xerostomia^15-18^. A study of dental students found that tobacco users (29.3%), e-cigarette users (33.1%), and dual users (28.1%), reported a significantly higher prevalence of xerostomia than non-smokers (23.4%)^19^. Chaffee et al.^20^ found that frequent/always dry mouth was more prevalent among frequent (>5 days/month) e-cigarette (14%) and combustible tobacco users (19%). E-cigarette users, including 14.9% of US adolescents aged 13–17 years and 24.4% of medical students, reported dry mouth^9-21^. Among e-cigarette users aged ≥18 years, the prevalence of xerostomia was 31.0%^8^. Smokers were more likely to have dry mouths in all ages and sexes^22^.

The relationship between smoking and xerostomia is crucial for public health. Smoking harms nearly every organ in the body. Severe health effects include the risk of nicotine addiction and potential harm to brain development from e-cigarette use^23^, as well as potential respiratory and cardiovascular hazards associated with e-cigarette use^24^. As the potential association of combustible tobacco and e-cigarette use with xerostomia has not been fully explored, this systematic review and meta-analysis sought to provide an evidence-based estimation of the global prevalence of xerostomia among healthy people who used e-cigarettes and combustible tobacco.

## METHODS

We conducted a meta-analysis of observational studies comparing the prevalence of xerostomia in healthy populations who smoke. This meta-analysis was performed according to the PRISMA (Preferred Reporting Items for Systematic Reviews and Meta-Analyses) guidelines. The meta-analysis has been registered on the International Prospective Register of Systematic Reviews (PROSPERO), and the trial registration number is CRD42022319706.

### Search strategy

The following electronic databases were searched in this study: Web of Science, PubMed, Cochrane Library, Embase, Chinese Biomedical Literature Database (CBM), Chinese National Knowledge Infrastructure (CNKI), China Science and Technology Journal Database (VIP), and Wan-Fang Database from 1 January 2000 to 20 October 2022, independently by two researchers (XG and PX). There were no limitations on publication status, but the language was limited to Chinese and English. Using the terms: ‘dry mouth’, ‘xerostomia’, ‘cigarette smoking’, ‘tobacco’, ‘cigarette smoking’, ‘smoking’, ‘Electronic Nicotine Delivery Systems’ and ‘e-cigarettes’, articles were searched in all available combinations. The complete search strategy is available in the Supplementary file.

### Selection criteria

Xerostomia was defined as the subjective sensation of dry mouth or low salivary flow rates. The inclusion criteria were: 1) focus on healthy people; 2) include data on the prevalence of xerostomia in e-cigarette and/or combustible tobacco users; and 3) observational studies. The researchers excluded articles that: 1) were duplicate publications, reviews, case reports, incomplete, incorrect information, or low-quality evaluation; 2) had restricted access to the full text; 3) were in a language other than English and Chinese.

### Extraction of data

Included articles were independently reviewed by two researchers (XG and XP), based on title/abstract first, and full-text second, according to the inclusion criteria. Any controversy was resolved by a discussion or with a third researcher (LH). The following information was extracted using a defined standard data template form: study characteristics, country, participants, and results. Two reviewers (XG and TF) independently extracted and verified data using the template, and any disagreements were settled through discussion.

### Bias and quality assessment

The Newcastle-Ottawa Scale (NOS) was used to assess the quality of the literature of case-control studies^25^, which evaluates the quality of the literature in terms of study population selection, comparability, exposure (case-control studies), or outcome (cohort studies), with a score of 0–9. Besides, we used quality evaluation criteria for cross-sectional studies recommended by the Agency for Healthcare Research and Quality (AHRQ). The proposed criteria include 11 items, with one point for each item, and the quality of the literature is based on the total score, which is divided into low (1–3), medium (4–7), and high quality (8–11)^26^.

### Statistical analysis

Stata 15.0 software was used for single-group rate meta-analysis. The I^2^ statistic detected statistical heterogeneity. The random-effect model was used for data synthesis. Significant clinical heterogeneity was addressed using subgroup analysis. Subgroup analysis factors included type of smoking, country, and age. The potential publication bias of the meta-analysis was determined by Begg’s test and Egger’s test.

## RESULTS

**Figure 1:**
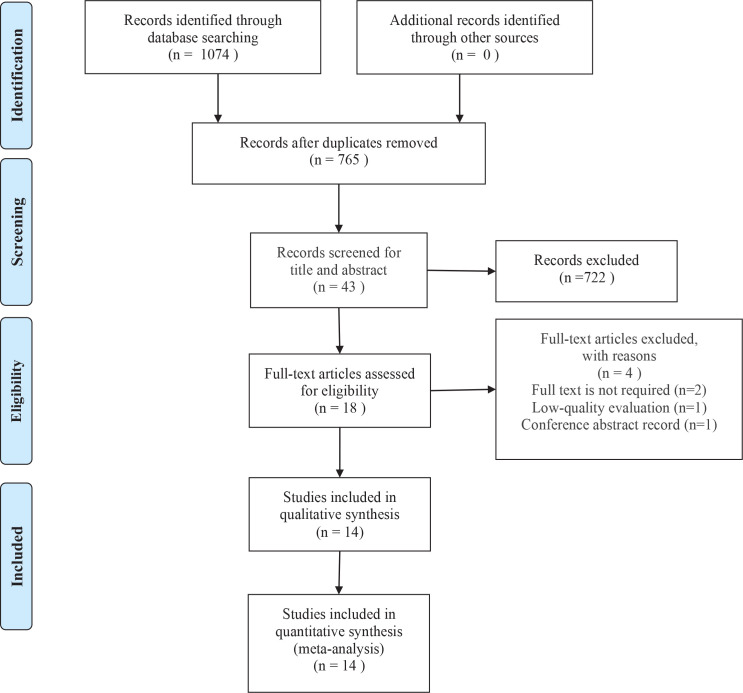
Flow diagram of the study selection process

In total, 1074 published articles were collected from online databases, including Web of Science, PubMed, Cochrane Library, Embase, Chinese Biomedical Literature Database (CBM), Chinese National Knowledge Infrastructure (CNKI), Chinese Scientific Journal Database (VIP), and Wan-Fang Database. All studies were published from 1 January 2000 to 20 October 2022. After deleting duplicates, 765 studies were obtained. After the review of the titles and abstracts, 43 articles were chosen for full-text review. Due to the unavailability of full text or not measuring the outcome of interest, 14 articles were selected for the qualitative synthesis. Finally, 14 articles^8,9,19-21,25-33^ were included in the quantitative synthesis (meta-analysis), including 12 cross-sectional and 2 case-control studies. The PRISMA flow diagram of study selection is shown in Figure 1.

### Characteristics and risk of bias evaluation of the included studies

The characteristics of the included studies can be found in Table 1. Data were extracted on the author, year of publication, country, study design, sample size (cases), the prevalence of xerostomia, age, gender, outcome (s) reported, and definition of smokers. The year of publication of the included literature was between 2014 and 2022. We identified studies across multiple countries: USA (n=3), Saudi Arabia (n=3), Pakistan (n=2); and one study in each of India, Poland, Italy, China, Egypt, plus a study with participants from 11 countries (Croatia, Iraq, Jordan, Kuwait, Lebanon, Malaysia, Nigeria, Saudi Arabia, South Africa, Turkey, and Yemen). Overall, 6827 participants were included in this study from all articles. The sample size of studies ranged from 39 to 1624 participants. Type of smoking includes both combustible tobacco and e-cigarettes. The age ranges were between 14 and 65 years. The included studies reported males to be the majority of their participants. The quality appraisal of the included studies is shown in Tables 2 and 3.

**Table 1:** Characteristics of the included studies on the prevalence of xerostomia among e-cigarette or combustible tobacco users

*Authors Year*	*Study design*	*Country*	*Sample size (Cases)*	*Prevalence of xerostomia*	*Age (years) Mean ± SD*	*Gender*	*The outcome(s) reported*	*Definition of smokers*
Dyasanoor et al.^25^ 2014	Case-control	India	Combustible tobacco: 60	37.00%	36.98 ± 11.52	NR	Modified Schirmer test and PROs	Daily smoking for six months
Ullah et al.^26^ 2015	Cross-sectional	Pakistan	E-cigarettes/combustible tobacco: 250	10.40%	21.66 ± 2.07	100% male	PROs	NR
King et al.^8^ 2019	Cross-sectional	USA	E-cigarettes: 1624	31.00%	18–24: 25.5% 25–44: 48.2% ≥45: 26.3%	56.1% male 43.9% female	PROs	Ever e-cigarette users
Kumar et al.^27^ 2019	Cross-sectional	Pakistan	E-cigarettes/combustible tobacco: 377	15.12%	aged 28–64	90.45% male 9.55% female	PROs	NR
Lewek et al.^28^ 2019	Cross-sectional	Poland	E-cigarettes: 1032	8.30%	25.9 ± 11.1	85.6% male 14.4% female	PROs	Ever smoked or currently smoking
Gallus et al.^29^ 2020	Cross-sectional	Italy	E-cigarettes: 395	25.40%	51.3 ± 13.0	53.6% male 46.4% female	PROs	Current and ex-smokers
Habib et al.^21^ 2020	Cross-sectional	Saudi Arabia	E-cigarettes: 49	24.49%	aged 18–23	NR	PROs	Ever smoked or currently smoking
Nazir et al.^30^ 2020	Cross-sectional	Saudi Arabia	E-cigarettes/combustible tobacco: 199	31.20%	<16	NR	PROs	NR
King et al.^9^ 2020	Cross-sectional	USA	E-cigarettes/combustible tobacco: 141	14.18%	16	60.2% male 39.8% female	PROs	Past 30-day smoking
Chaffee et al.^20^ 2021	Cross-sectional	USA	E-cigarettes: 976	Occasionally: 54.2% Frequently: 5.4% Always: 0.4%	<18	36.6% male 60.9% female 2.6% other	PROs	Past 30-day smoking
Peng et al.^31^ 2021	Case-control	China	E-cigarettes: 85 Combustible tobacco: 92	E-cigarettes: 35.29% Combustible tobacco: 8.69%	E-cigarettes: 21.87 ± 1.79 Combustible tobacco: 21.66 ± 2.36	E-cigarettes: 65.88% male 34.12% female Combustible tobacco: 58.7% male 41.3% female	PROs	History of smoking ≥1 year
Kabbash et al.^32^ 2022	Cross-sectional	Egypt	E-cigarettes: 39	48%	NR	NR	PROs	NR
Tantawi et al.^33^ 2022	Cross-sectional	Saudi Arabia	E-cigarettes/combustible tobacco: 657	18.60%	18–23	NR	PROs	NR
Alhajj et al.^19^ 2022	Cross-sectional	11 countries: Croatia, Iraq, Jordan, Kuwait, Lebanon, Malaysia, Nigeria, Saudi Arabia, South Africa, Turkey, and Yemen	E-cigarettes: 255 Combustible tobacco: 596	E-cigarettes: 31.76% Combustible tobacco: 28.86%	E-cigarettes: ≤20 : 72 >20 : 183 Combustible tobacco: ≤20 : 178 >20 : 418	E-cigarettes: 65.88% male 30.98% female Combustible tobacco: 57.72% male 42.28% female	PROs	NR

NR: not reported. PROs: patient-reported outcomes.

**Table 2:** Results of the risk of bias evaluation for case-control studies included (score)

*Study*	*Selection*	*Comparability*	*Exposure*	*Score*
Diasakos et al. 2014	4	1	2	7
Peng et al. 2021	4	2	1	7

**Table 3:** Risk of bias evaluation results for cross-sectional studies included (score)

*Study*	*①*	*②*	*③*	*④*	*⑤*	*⑥*	*⑦*	*⑧*	*⑨*	*⑩*	*⑪*	*Score*
Ullah et al. 2015	1	1	1	1	1	0	1	0	0	1	0	7
King et al. 2019	1	1	1	1	0	1	1	1	0	1	0	8
Lewek et al. 2019	1	1	1	1	0	1	1	0	1	1	0	8
Kumar et al. 2019	1	1	1	1	1	1	1	1	0	1	0	9
King et al. 2020	1	1	1	1	1	0	1	1	0	1	0	8
Habib et al. 2020	1	1	1	1	1	1	1	0	0	1	0	8
Nazir et al. 2020	1	1	1	1	1	1	1	1	0	1	0	9
Gallus et al. 2020	1	1	1	1	0	1	1	1	0	1	0	8
Chaffee et al. 2021	1	1	1	1	1	1	1	1	1	1	1	11
Alhajj et al. 2022	1	1	1	1	1	1	1	1	0	1	0	9
Tantawi et al. 2022	1	1	1	1	0	1	1	1	1	1	0	9
Kabbash et al. 2022	1	1	1	1	1	1	1	0	1	1	0	9

① Define the source of information (survey, record review);

② List inclusion and exclusion criteria for exposed and unexposed subjects (cases and controls) or refer to previous publications;

③ Indicate time period used for identifying patients;

④ Indicate whether or not subjects were consecutive if not population-based;

⑤ Indicate if evaluators of subjective components of study were masked to other aspects of the status of the participants;

⑥ Describe any assessments undertaken for quality assurance purposes (e.g. test/retest of primary outcome measurements);

⑦ Explain any patient exclusions from analysis;

⑧ Describe how confounding was assessed and explain any patient exclusions from analysis;

⑨ If applicable, explain how missing data were handled in the analysis;

⑩ Summarize patient response rates and completeness of data collection;

⑪ Clarify what follow-up, if any, was expected and the percentage of patients for which incomplete data or follow-up was obtained.

### The prevalence of xerostomia

**Figure 2:**
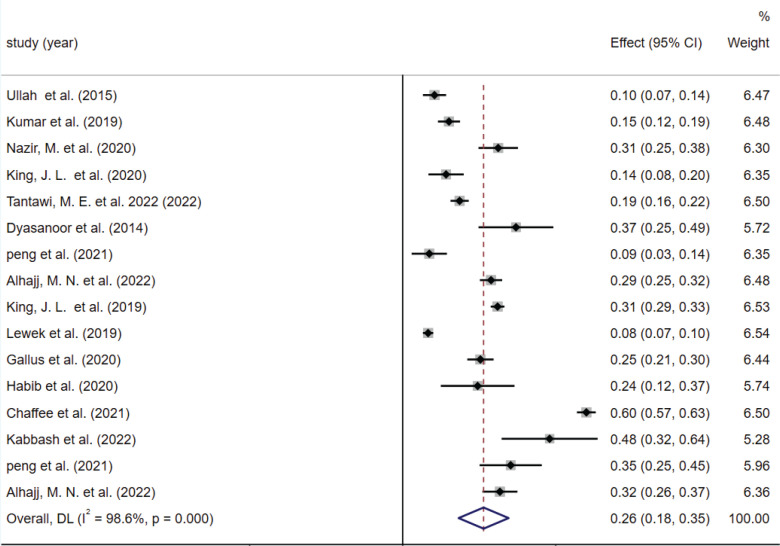
Forest plot of the overall included studies on the prevalence of xerostomia among e-cigarette or combustible tobacco users

A total of 14 studies were included ^8,9,19-21,25-33^, with a total sample size of 6827 cases. We summarized the prevalence of xerostomia for all smokers. Two studies^25,31^ reported the prevalence of xerostomia in both e-cigarette and combustible tobacco populations, and we included these two indexes in meta-analyses to synthesize the data separately. The results of the random-effects model meta-analysis showed that the overall prevalence of xerostomia was 26% (95% CI: 18–35) in the population of smokers (combustible and/or e-cigarette) (Figure 2). The published risk of bias assessment indicated that the risk for publication bias was not statistically identified by the Begg test (p=0.499), but was detected by the Egger test (p=0.007). We present publication bias plots in Supplementary file Material 1.

### Subgroup analysis

**Figure 3:**
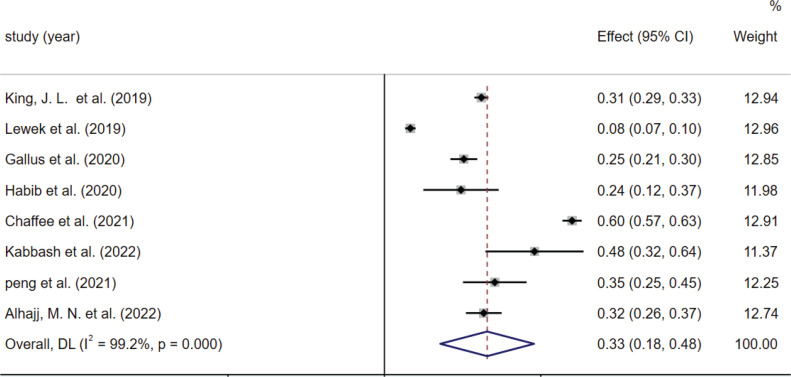
The effect size and 95% CI of included studies based on e-cigarette use

**Figure 4:**
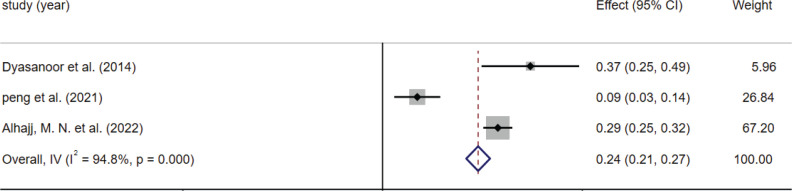
The effect size and 95% CI of included studies based on combustible tobacco use

Subgroup analysis was performed according to the type of smoking (Figures 3 and 4), country, and age. The results differed by the type of smoking: the prevalence of dry mouth was 33% (95% CI: 18–48) in the e-cigarette population and 24% (95% CI: 21–27) in the combustible tobacco population. Results also differed by country: the prevalence of xerostomia was 39% (95% CI: 37–40) in the United States, 13% (95% CI: 10–15) in Pakistan, and 21% (95% CI: 18–24) in Saudi Arabia. When the included literature was grouped according to age: the prevalence of dry mouth was 30% (95% CI: 28–32) for those aged <25 years and 20% (95% CI: 18–23) for 25–65 years (Table 4). We presented forest plots of subgroup analyses in Supplementary file Material 2.

**Table 4:** Subgroup analyses on the prevalence of xerostomia among e-cigarette or combustible tobacco users

*Variables*	*Number of studies*	*% (95% CI)*	*p*	*Heterogeneity*	
				*I^2^ (%)*	*p*
**All studies**	14	26 (18–35)	<0.001	99	<0.001
**Type of smoking**					
E-cigarette	8	33 (18–48)	<0.001	99	<0.001
Combustible tobacco	3	24 (21–27)	<0.001	95	<0.001
**Country**					
USA	3	39 (37–40)	<0.001	99	<0.001
Saudi Arabia	3	21 (18–24)	<0.001	84	0.002
Pakistan	2	13 (10–15)	<0.001	68	0.077
**Age** (years)					
<25	6	30 (28–32)	<0.001	99	<0.001
25–65	3	20 (18–23)	<0.001	90	<0.001

## DISCUSSION

This systematic review was performed to evaluate the prevalence of xerostomia in healthy populations with e-cigarettes and/or combustible tobacco and assess the influencing factors of dry mouth. The included literature included 12 cross-sectional studie^8,9,19-21,26-30,32,33^ and 2 case-control studies^25,31^. We sought to explore the influences of smoking on xerostomia to help better understand the oral health risks of smoking and support healthcare providers in planning viable cessation programs that would help smokers effective and long-term cessation.

Subgroup analysis found significant differences in xerostomia by smoking type, country, and age. The difference for us is that the prevalence of dry mouth is higher for e-cigarettes (33%) than for combustible tobacco (24%), which follows the findings of Peng et al.^31^ and Alhajj et al.^19^. In previous reports, e-cigarettes were considered less harmful to users as an aid to smoking cessation and a replacement for regular cigarettes^28,34,35^. Pop et al.^36^ found that clinically healthy young tobacco smokers and e-cigarette users presented an increased number of micronuclei in the oral epithelial cells, compared to non-smoking individuals. Bardellini et al.^37^ reported e-cigarette users to have more frequent oral lesions, burns, or inflammation compared to former or non-smokers. Since aerosol propylene glycol and glycerin are the main components of e-liquid, they are associated with mouth and throat irritation. The adverse effects most frequently mentioned by those surveyed (such as a sore/dry throat, cough, and mouth/throat discomfort) are frequently described in the literature^38,39^.

In addition, the prevalence of xerostomia was higher in the United States (39%) than in Pakistan (13%) and Saudi Arabia (21%). More than half of the US adults in a large, national sample who had ever used an e-cigarette reported experiencing at least one negative symptom as a result of their usage. Dry mouth is one of the most common symptoms^8^. Moreover, smokers under 25 years of age (30%) were more likely to report dry mouth, which is similar to the findings of King et al.^9^. Because of the high level of self-consciousness among young people, e-cigarette use is growing in popularity. Young adults aged 18–24 years were found to have the highest prevalence of smoking, as were both current and former smokers^40^. In an analysis based on the Eurobarometer 385 survey of 26566 participants, it was found that respondents aged 15–24 years were 3.3 times more likely to be users versus older participants^41^.

It is worth mentioning that there is no uniform definition of smokers and assessment of xerostomia, in all the literature. We summarized the definition of smoking: ‘Ever smoked or currently smoking’, ‘Past 30-day smoking’, ‘History of smoking ≥ 1 year’, and ‘Daily smoking for six months’. All included studies defined xerostomia as a subjective patient-reported sensation of dry mouth, and this leads to differences in the prevalence of xerostomia. Chaffee et al.^20^ categorized dry mouth as ‘Occasionally’, ‘Frequently’, and ‘Always’, which makes the prevalence of dry mouth as high as 60%. While perception is important for understanding, clinical validation may be needed to verify symptoms and determine the severity of symptoms when used. Furthermore, e-cigarette and tobacco products differ significantly, and different product characteristics may be associated with different types and frequencies of symptoms.

### Strengths and limitations

To the best of our knowledge, this study is the first meta-analysis to evaluate the prevalence of xerostomia among e-cigarette and/or combustible tobacco users. This review included studies with 6827 participants. Moreover, we also performed subgroup analyses to discuss the prevalence of xerostomia by smoking type, country, and age.

However, this study also has some limitations. First, most of the included studies were short-term observational studies, so some biases could not be avoided due to the limitations of the study design. Moreover, after doing a subgroup analysis, the heterogeneity among various subgroups remained significant, and the variables driving research heterogeneity could not be determined, which may impair the correctness of the results. Furthermore, the subgroup analyses were limited to three characteristics, but future studies could explore additional characteristics such as study setting, the participant’s education level, socioeconomic status, smoking habits, and severity of dry mouth. Finally, the number of studies included in the analyses was limited, needing the inclusion of more high-quality studies to offer evidence.

## CONCLUSIONS

Research shows that smokers may be more likely to experience dry mouth. E-cigarette users were more likely to have dry mouth than tobacco users. Dry mouth is more common among young smokers, which suggests that we should perhaps pay more attention to the awareness of the risks of smoking among young people and take appropriate measures to aid cessation. However, more research into the association between smoking and the severity of dry mouth is needed. Because the number of smokers has increased in recent years, and dry mouth can negatively impact people’s quality of life, oral hygienists should give more emphasis to tobacco and e-cigarette use in clinical practice. The above conclusions need to be verified by additional high-quality studies due to the constraints of the number and quality of the included studies.

## Data Availability

The data supporting this research can be found in the Supplementary file.
