# Corrigendum: The prevalence of xerostomia among e-cigarette or combustible tobacco users: A systematic review and meta-analysis

**DOI:** 10.18332/tid/161989

**Published:** 2023-03-27

**Authors:** Xingtong Guo, Lili Hou, Xuepei Peng, Fuyou Tang

**Affiliations:** 1 School of Nursing, Chengdu University of Traditional Chinese Medicine, Chengdu, China; 2 Nursing Department, Shanghai Ninth People’s Hospital, Shanghai Jiaotong University School of Medicine, Shanghai, China

**Keywords:** e-cigarettes, meta-analysis, tobacco, xerostomia

Corrigendum on:

The prevalence of xerostomia among e-cigarette or combustible tobacco users: A systematic review and meta-analysis

By Xingtong Guo, Lili Hou, Xuepei Peng, Fuyou Tang

Tobacco Induced Diseases, Volume 21, Issue February, Pages 1–11, Publish date: 9 February 2023

DOI: https://doi.org/10.18332/tid/156676

In the originally published version of the article, on pages 1, 5, and 7, the expression 95% CI: 21–17 should read 95% CI: 21–27. This is corrected also online.

