# Supplementary material for: The prevalence of xerostomia among e-cigarette or combustible tobacco users: A systematic review and meta-analysis
Source: Tob Induc Dis. 2023 Feb 9;21:22. doi: 10.18332/tid/156676 (PMC9909684; doi:10.18332/tid/156676)
Supplement: Supplementary file 1 [file TID-21-22-s1.pdf]

Search strategy to be used in PubMed	
Search line	Search items
#1	Xerostomia [Mesh]
#2	Xerostomia
#3	dry mouth
#4	#1 OR #2 OR #3
#5	Cigarette Smoking [Mesh]
#6	Tobacco [Mesh]
#7	Cigarette Smoking
#8	Smoking
#9	Tobacco
#10	Smoker
#11	#5 OR #6 OR #7 OR #8 OR #9 OR #10
#12	Electronic Nicotine Delivery Systems [Mesh]
#13	Electronic Nicotine Delivery Systems
#14	e-cigarettes
#15	#12 OR #13 OR #14
#16	#11 OR #15
#17	Incidence [Mesh]
#18	Prevalence [Mesh]
#19	Correlation of Data [Mesh]
#20	Incidence
#21	Correlation
#22	Prevalence
#23	Attack Rate
#24	#17 OR #18 OR #19 OR #20 OR #21 OR #22 OR #23
#25	#4 AND #16 AND #24 AND (humans[Filter])

Search strategy to be used in Embase	
#1	xerostomia'/exp OR 'xerostomia'
#2	('cigarette'/exp OR cigarette) AND ('smoking'/exp OR smoking)
#3	tobacco'/exp OR tobacco
#4	electronic AND ('cigarette'/exp OR cigarette)
#5	people by smoking status'/exp OR 'people by smoking status'
#6	#2 OR #3 OR #4 OR #5
#7	'prevalence'/exp OR prevalence
#8	'incidence'/exp OR incidence
#9	('correlation'/exp OR correlation) AND ('analysis'/exp OR analysis)
#10	correlational study'/exp OR 'correlational study'
#11	#7 OR #8 OR #9 OR #10
#12	#1 AND #6 AND #11
#13	#1 AND #6 AND #11 AND ([chinese]/lim OR [english]/lim)

Search strategy to be used in Cochrane	
#1	incidence OR prevalence OR correlation OR "attack rate"
#2	Cigarette Smoking OR Tobacco OR "Electronic Nicotine Delivery Systems" OR Smoking OR e-cigarettes
#3	"dry mouth" OR xerostomia
#4	#1 AND #2 AND #3
URL:Advanced Search | Cochrane Library	

Search strategy to be used in Wos	
#1	TS=(Xerostomia OR (dry mouth))
#2	TS=((Cigarette Smoking) OR Tobacco OR Smoking OR Smoker OR (Electronic Nicotine Delivery Systems) OR e-cigarettes)
#3	TS=(incidence OR prevalence OR correlation OR (Attack Rate))
#4	#1 AND #2 AND #3
URL:https://www.webofscience.com/wos/alldb/summary/07153490-6316-4171-ae07-fddb1d14b87e-5ad55a45/relevance/1	

Search strategy to be used in Chinese National Knowledge Infrastructure (CNKI)	
	Keyword and Abstract:
#1	电子烟 + 吸烟 + 烟草 + 香烟 + 尼古丁
#2	口干 + 口腔干燥 + 口干症
#3	发生率 + 率
#4	#1 AND #2 AND #3
https://kns.cnki.net/kns8/AdvSearch?dbprefix=CFLS&&crossDbcodes=CJFQ%2CCDMD%2	

Search strategy to be used in China Science and Technology Journal Database(VIP)	
	检索式检索
((((((题名或关键词=电子烟 OR 题名或关键词=吸烟) OR 题名或关键词=烟草) OR 题名或关键词=香烟) OR 题名或关键词=烟) AND ((题名或关键词=口干 OR 题名或关键词=口腔干燥) OR 题名或关键词=口干症)) AND (题名或关键词=发生率 OR 题名或关键词=率))	
高级检索-【维普期刊官网】- 中文期刊服务平台 (cqvip.com)	

Search strategy to be used in Wan-Fang Database	
文献类型	期刊论文、学位论文、中外标准
检索信息	题名或关键词/模糊
#1	电子烟 OR 吸烟 OR 烟草 OR 香烟 OR 烟
#2	口干 OR 口腔干燥 OR 口干症
#3	#1 AND #2
万方数据知识服务平台 (wanfangdata.com.cn)	

Search strategy to be used in Chinese Biomedical Literature Database (CBM)	
检索条件	("口腔干燥"[不加权:扩展]) AND ((((("吸烟"[不加权:扩展]) OR "烟草"[不加权:扩展]) OR "电子烟"[不加权:扩展]) OR "尼古丁"[不加权:扩展]) OR "烟草制品"[不加权:扩展])
URL	http://www.sinomed.ac.cn/index.jsp

Supplementary Material 1.

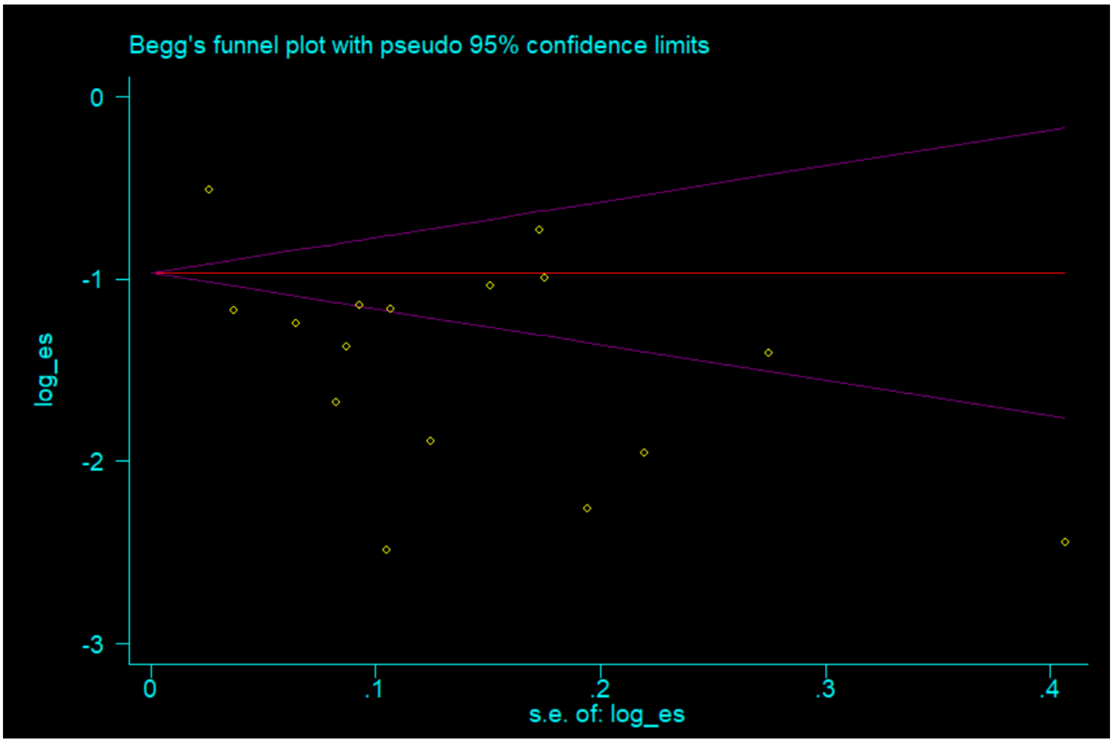


```
Begg's Test

  adj. Kendall's Score (P-Q) =        16
          Std. Dev. of Score =     22.21
           Number of Studies =        16
                           z =      0.72
                    Pr > |z| =     0.471
                           z =      0.68 (continuity corrected)
                    Pr > |z| =     0.499 (continuity corrected)
```

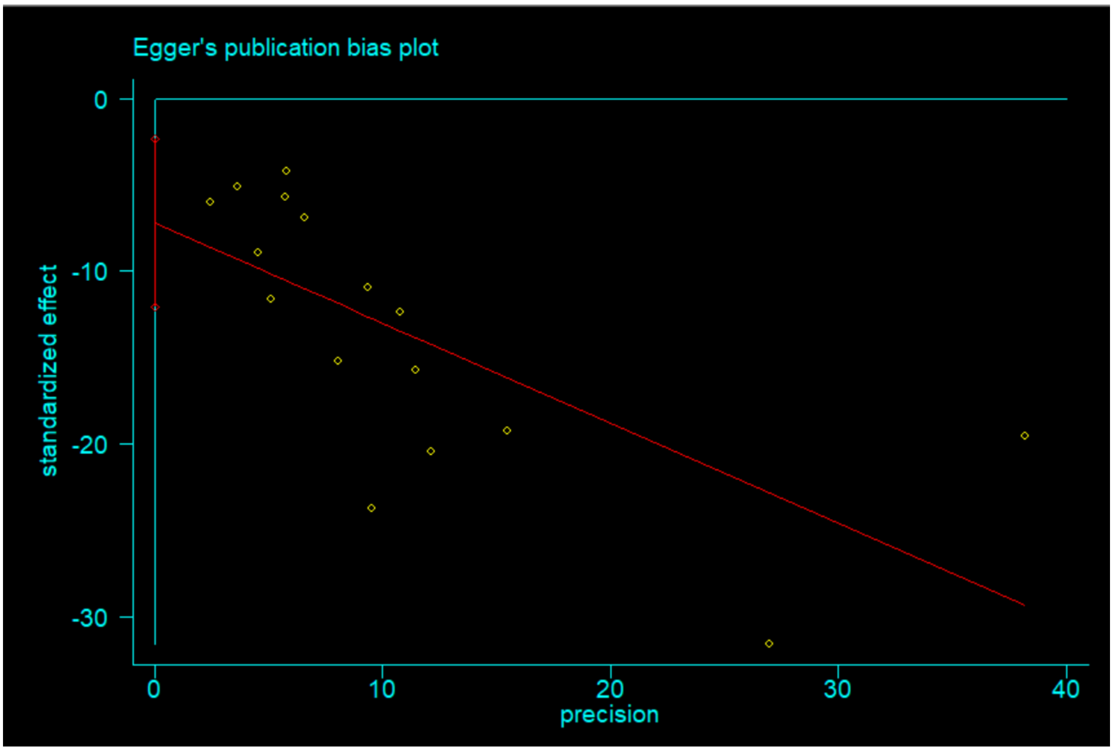

Egger's test

Std_Eff	Coef.	Std. Err.	t	P>|t|	[95% Conf.	Interval]
slope	-.5782297	.1598537	-3.62	0.003	-.9210818	-.2353776
bias	-7.214132	2.275731	-3.17	0.007	-12.09509	-2.333175

Supplementary Material 2 Forest Plot of Subgroup Analyses

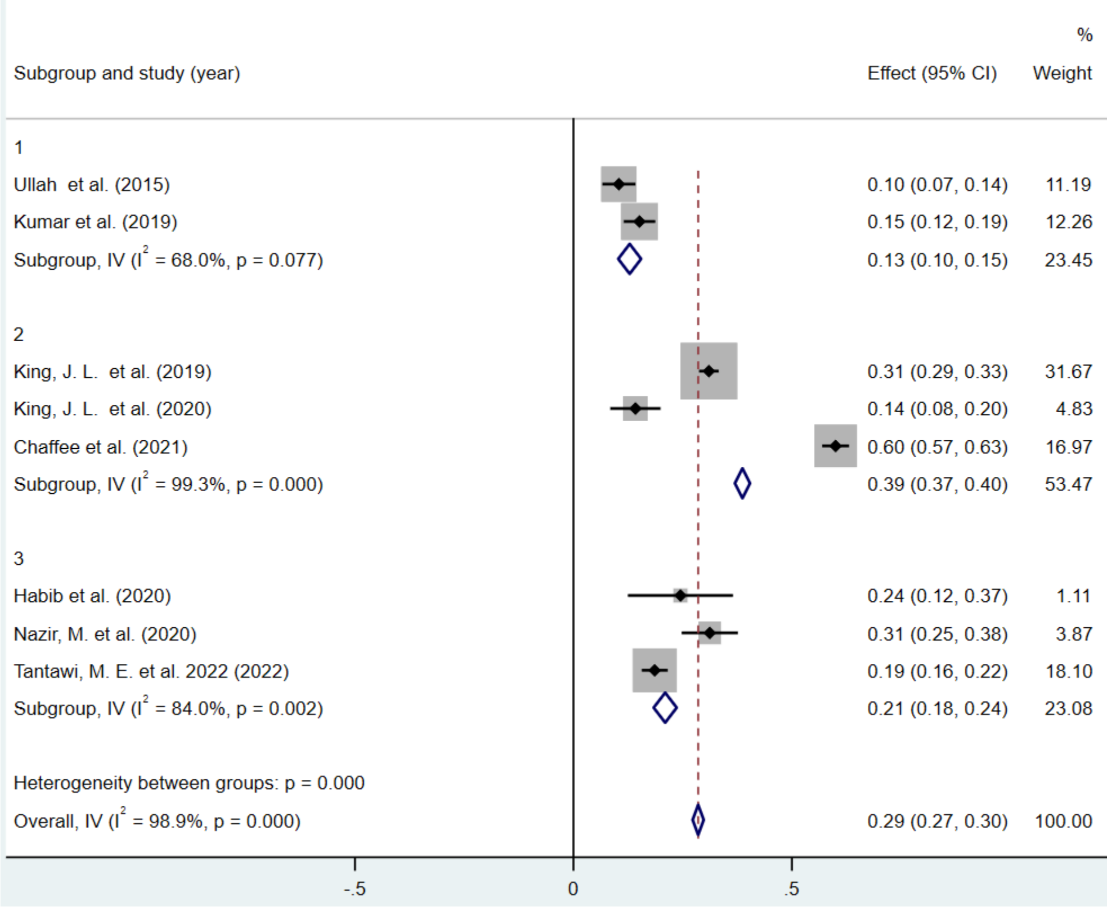

Fig 1. The effect Size and 95%CI of included studies based on Country

1: Pakistan; 2:American; 3: Saudi Arabia.

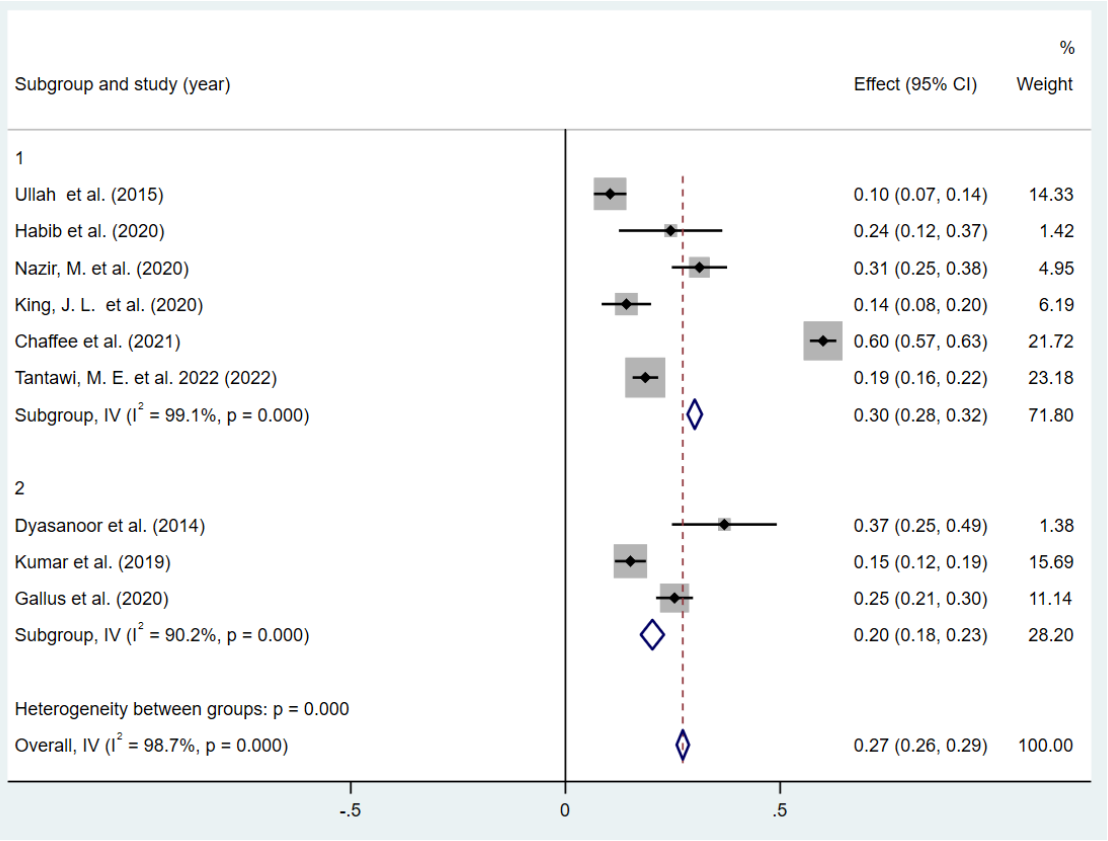

Fig 2. The effect Size and 95%CI of included studies based on Age
1:≤25; 2:25-65

© 2023 Guo X. et al.
